# Resilient managed competition during pandemics: lessons from the Italian experience

**DOI:** 10.1017/S1744133120000365

**Published:** 2020-09-04

**Authors:** Germà Bel, Marc Esteve

**Affiliations:** 1 Universitat de Barcelona, Barcelona, Spain; 2 University College London, London, UK

## Abstract

One of the main governance decisions that policymakers need to make is whether to implement public services via centralized or decentralized forms. As Costa *et al*. discuss in their article, when public services are implemented via competing systems, service providers contend to provide good services with the ultimate objective of gaining market quota. This is known as managed competition (MC), as the authorities will have to manage the panoply of public and private organizations offering the service. The alternative is to manage the service more centrally, in what it is identified as vertical integration. As the authors describe, several governments around the globe have abandoned their vertical integrated models in favour of decentralized models. This is the case, as the authors recall, for most health services in Europe. While there is an emerging body of evidence suggesting that decentralized MC outperforms vertically integrated models both in terms of efficiency and in terms of service quality, little is known on how these systems react under different circumstances. This means, for example, how these systems can cope with a sudden increase in their service demands.

One of the main governance decisions that policymakers need to make is whether to implement public services via centralized or decentralized forms. As Costa *et al*. discuss in their article, when public services are implemented via competing systems, service providers contend to provide good services with the ultimate objective of gaining market quota. This is known as managed competition (MC), as the authorities will have to manage the panoply of public and private organizations offering the service. The alternative is to manage the service more centrally, in what it is identified as vertical integration. As the authors describe, several governments around the globe have abandoned their vertical integrated models in favour of decentralized models. This is the case, as the authors recall, for most health services in Europe. While there is an emerging body of evidence suggesting that decentralized MC outperforms vertically integrated models both in terms of efficiency and in terms of service quality, little is known on how these systems react under different circumstances. This means, for example, how these systems can cope with a sudden increase in their service demands.

Costa *et al*. identify the current Covid-19 pandemic as an opportunity to assess how health services operating under vertically integrated models compare to MC initiatives when dealing with an unforeseen upsurge of service demands. In a nutshell, the authors provide empirical evidence of the results of a stress test in the health system. Their main argument is that MC organizational models based on MC without an integrated authority do not face the incentives either to cooperate with other service providers or to offer a rapid reaction. This, according to Costa *et al*., can, therefore, result in a delayed response and, ultimately, in higher mortalities.

In our view, the article presents two core strengths. The first one is that the authors provide a well-crafted review of both centralized and decentralized models of MC. The second is that they have gathered their empirical evidence from Italy, a very suitable country because it has both centralized and decentralized MC systems operating in different regions, and also because, sadly, it has suffered greatly from Covid-19. In particular, the authors compare regions such as Emilia Romagna and Veneto, which operate under a more centralized model, with other regions that have opted for decentralized MC systems, such as Lombardy.

Costa *et al*. focus on the number of deaths per region, together with swab tests performed, to compare how each system has responded to the pandemic. According to their evidence, those regions operating with decentralized MC systems have seen the largest deaths ratio per inhabitant and a comparatively small frequency of swab tests. From which they conclude that decentralized MC systems may offer efficiency gains in normal times, but when the systems face a sudden increase in their demand as the one caused in an epidemic they seem to underperform those systems that rely on vertical integration. In the paragraphs below, we offer a critical analysis of these arguments, challenging some of the empirical evidence and delving further into the discussion of how to compare decentralized and centralized health care systems.

Different degrees of severity of Covid-19 consequences are likely explained by a variety of factors that have concurrent effects. Hence, trying to isolate effects from one single factor, such as the degree of vertical integration of the health system, is a hard task, because many other factors can be at play. One way to assess whether there is, generally, a correlation between health management systems and outcomes (such as fatality rates or tests performed) is to compare health systems with different management across relatively comparable jurisdictions. To do so, we have used the typology of health management systems and the corresponding classification of countries from OECD (2010). Then we have used data on the number of deaths per million inhabitants from Roser *et al*. (2020), publicly available at Our World in Data. We have computed data for each country, 67 days after the first death from Covid-19 was registered (which is the time lag corresponding to data on Italy from 30 April – first death recorded on 23 February). Figure 1 presents the results we have obtained.

**Figure 1.**
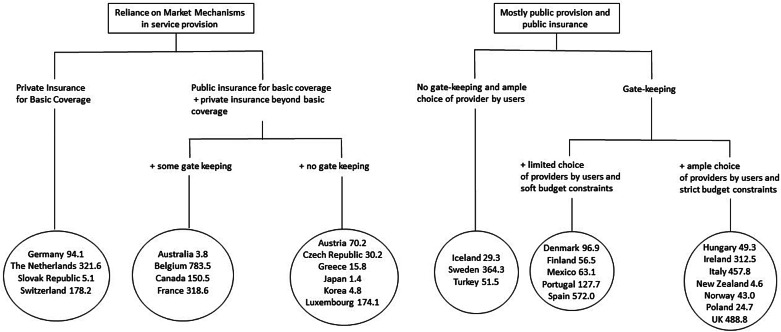
Groups of countries sharing broadly similar health systems. *Note*: Displayed values represent the deaths per million for each country (67 days after the first death was registered). The USA had 221.8 deaths per million.

These data do not allow us to establish any sensible correlation between health management systems and the severity of fatalities. The average death toll for systems based on reliance on the market (153.7 per million) is lower than that for systems based on public provision (182.8 per million). However, these numbers are hardly comparable; not only because the average numbers for each subcategory are divergent, but also so is the variance within each subcategory. Furthermore, the data above do not take into account the estimation of excess deaths, which are very different between countries. Hence, that data do not allow us to make robust claims that systems based on market reliance performed better. But in no way do they support the opposite: that is, that systems based on publicly controlled mechanisms (with a higher or lower degree of vertical integration) performed better.

A less generalizable but perhaps more fine-grained comparison with Italy can be obtained from analysing a single system that is relatively comparable to the Italian one. In this regard, we believe that the Spanish case meets those requirements well. First, it has a similar health management system to Italy – with regional management based on public provision, albeit with more limited room for choice of providers by users. Second, it is one of the two most similar countries (together with the UK) regarding the frequency of fatalities. Although there is not a specific database providing information on autonomous public and autonomous private hospitals in all Spanish regions, we have been able to construct variables on the weight of beds where management is vertically integrated (managed by hospitals directly controlled by the regions) and autonomous (here we could not make the distinction between public and private hospitals).

Table 1 presents data for all Spanish regions, ordered according to the number of deaths per million inhabitants on 11 May. Again, we cannot see any correlation between vertical integration of management and lower severity, or higher testing. In this case, three out of the four regions with a higher fatality rate are also among those with higher vertical integration. Again, no robust claims on a relationship between vertical integration and more fatalities can be made based on these data (recall that fatalities are relatively higher than in Italy, and so are excess deaths), but for sure the data are not consistent with the opposite claim. Nor is there a relationship between the degree of vertical integration and testing activity.

**Table 1.** Health management characteristics and outcomes in Spain

	Bed/100k	Vertically integrated (%)	Autonomous hospitals (%)	Death/million	PCR/1000 hab
Castilla La Mancha	256.4	94.5	5.5	1362.1	62.3
Madrid	217.7	90.6	9.4	1286.9	25.4
La Rioja	314.2	81.4	18.6	1095.2	22.9
Castilla León	297.1	100.0	0.0	793.3	46.9
Navarra	242.0	88.2	11.8	746.4	100.5
Catalunya	401.5	15.3	84.7	725.9	61.4
País Vasco	297.7	86.7	13.3	650.0	18.2
Aragón	338.9	89.8	10.2	620.1	38.7
Extremadura	340.7	86.5	13.5	454.9	57.1
Cantabria	253.1	92.1	7.9	345.1	73.2
Asturias	317.9	85.3	14.7	282.7	90.5
C. Valenciana	239.4	84.8	15.2	264.5	33.4
Galícia	308.3	90.4	9.6	219.4	80.5
Baleares	216.5	82.2	17.8	172.6	70.5
Andalucía	195.4	93.9	6.1	156.0	21.7
Murcia	232.5	95.1	4.9	92.4	89.5
Canarias	242.5	82.9	17.1	66.6	45.5

*Source*: (1) EPDATA base for total number of beds, and beds directly managed by SNS, and cumulated deaths in each region by 11 May (67th after the first death recorded in Spain) https://www.epdata.es/datos/hospitales-espana-datos-estadisticas/299

(2) Spanish Ministry of Health for percentage of beds included in the public care network (irrespective of titularity) http://inclasns.msssi.es/main.html?permalink=5d359b4afdd385c8c0013a8e1a63443d

(3) RTVE data on tests performed (as of 28th July 2020) https://www.rtve.es/noticias/20200728/mapa-del-coronavirus-espana/2004681.shtml

Certainly, on 14th March the Spanish Government did decree the State of Alarm, took over all relevant competencies (including health powers) on the crisis management, and nationalized private hospitals for Covid-19 management-related issues. Centralization of Covid-19-related purchases was chaotic and only 10 days after it had been decreed most regions went out to provider markets on their own, given the lack of provision by the central government.

Comparisons between vertically integrated and autonomous management of health systems in an emergency requires an extremely nuanced discussion. As pointed out by Christensen *et al*. (2016) a crisis emphasizes the need for strong leadership and central control at a strategic level on one side; but it also emphasizes the need for local autonomy and operational flexibility on the other. Local agility can be difficult if central restrictions are too strong and give limited room to local authorities. Hence, management systems must be decentralized to a certain point, which implies that politicians and public managers must facilitate a self-organized response system, instead of trying to control the system.

As empirical evidence emerges on this important new field of enquiry, new propositions and hypotheses will undoubtedly be developed on how decentralized should health systems be. The issues discussed by Costa *et al*. provide the initial outline for a future research agenda, which seeks to explore the effects of the Covid-19 pandemic outbreak across health systems. As governments seek new ways to deliver services and projects in times of fiscal austerity, studies that systematically examine how best to make a success of these organizational forms will undoubtedly be of enormous value.
